# Investigating causality in the association between vitamin D status and self-reported tiredness

**DOI:** 10.1038/s41598-019-39359-z

**Published:** 2019-02-27

**Authors:** Alexandra Havdahl, Ruth Mitchell, Lavinia Paternoster, George Davey Smith

**Affiliations:** 1 Medical Research Council Integrative Epidemiology Unit, University of Bristol, Bristol, BS8 2BN United Kingdom; 2 Population Health Sciences, Bristol Medical School, University of Bristol, Bristol, BS8 2BN United Kingdom; 3 Nic Waals Institute, Lovisenberg Diaconal Hospital, Oslo, 0853 Norway; 4 Department of Mental Disorders, Norwegian Institute of Public Health, Oslo, N-0213 Norway; 5 National Institute for Health Research Bristol Biomedical Research Centre, University Hospitals Bristol NHS Foundation Trust and University of Bristol, Bristol, United Kingdom

## Abstract

Self-reported tiredness or low energy, often referred to as fatigue, has been linked to low levels of circulating 25-hydroxyvitamin D (25OHD), a biomarker of vitamin D status. Although it is uncertain if the association is causal, fatigue is a common indication for testing, and correcting, low 25OHD-levels. We used two-sample Mendelian randomization to test for genetic evidence of a causal association between low 25OHD-levels and fatigue. Genetic-25OHD associations were estimated from the largest genome-wide association study of vitamin D to date, and genetic-fatigue associations were estimated in 327,478 individuals of European descent in UK Biobank, of whom 19,526 (5.96%) reported fatigue (tiredness or low energy nearly every day over the past two weeks). Using seven genome-wide significant 25OHD-reducing genetic variants, there was little evidence for a causal effect of 25OHD on fatigue (odds ratio for fatigue was 1.05 with 95% confidence interval of 0.87–1.27 per 1-SD decrease in log-transformed 25OHD). There was also little evidence of association between any individual 25OHD-reducing variant and fatigue. Our results suggest that a clinically relevant protective effect of 25OHD-levels on fatigue is unlikely. Therefore, vitamin D supplementation of the general population to raise 25OHD-levels is not likely to be useful in preventing fatigue.

## Introduction

Tiredness or low energy, often referred to as fatigue, is a common complaint in the general population and one of the leading reasons for consulting primary health care services^1–4^. Fatigue is a complex and nonspecific phenomenon. It is both a normal response to physical and mental exertion or stress, and a feature of illnesses such as infection and inflammation, multiple sclerosis, rheumatoid conditions, cancers, depression and anxiety^5^. In addition, fatigue is a defining characteristic of chronic fatigue syndrome/myalgic encephalomyelitis (CFS/ME), requiring that fatigue is profound and impairing, long-lasting, and medically unexplained^6^.

There is no generally accepted set of criteria for fatigue, and the prevalence of fatigue varies widely depending on the assessment method and criteria for severity and duration of tiredness or low energy. The cross-sectional prevalence of broadly defined fatigue in U.S. workers and home-living adults over the age of 50 was 37.9% (answered yes to having low levels of energy, poor sleep, or a feeling of fatigue during the past two weeks) and 31.2% (answered yes to feeling that everything was an effort or could not get going during the past week), respectively^1,3^. Of 15,283 individuals aged 18–45 years who were registered with general practices in England, 37.9% scored above the cutoff for substantial current fatigue on the widely used self-rated Chalder fatigue questionnaire^2^. A systematic review of case definitions for CFS/ME showed that cross-sectional prevalence ranged from less than 0.1% to 7.6%, depending on the criteria for duration and associated symptoms^7^. Findings suggest that self-reported fatigue is continuously distributed in the general population^2,8,9^, ranging from mild tiredness or low energy to debilitating exhaustion or weakness. Therefore, any boundary between normal and abnormal levels of fatigue may be arbitrary. Nevertheless, a threshold that has been used to classify clinically significant fatigue is self-reported tiredness or lack of energy on nearly every day or more over a period of at least two weeks^10^. Findings suggest that in the general population approximately 6% meet this criterion for current fatigue (feeling tired nearly all the time for more than two weeks during a one-month period)^11^.

One biological factor that has been linked to fatigue is vitamin D insufficiency (low levels of circulating 25-hydroxyvitamin D [25OHD])^12–18^, although findings have been mixed^19–22^. The association between fatigue and vitamin D insufficiency has attracted considerable attention in the popular press, and readers are encouraged to seek vitamin D testing and/or supplementation if feeling fatigued^23,24^. Studies show that patient-reported fatigue is one of the leading reasons for 25OHD testing^25,26^. Tiredness, fatigue or exhaustion was the reason for 22.4% of vitamin D test requests within the Northumbria Healthcare NHS Foundation Trust in England in 2017^26^.

Although the link between vitamin D status and fatigue has already begun to affect clinical practice, there is uncertainty as to whether the association is causal. In observational studies, the potential for reverse causality is substantial, as low vitamin D status could be a marker, or a consequence, of fatigue. Furthermore, the observed association between vitamin D status and fatigue could be spurious, simply reflecting unmeasured confounders that independently associate with lowered vitamin D status and fatigue. For example, vitamin D status and fatigue are both associated with a range of lifestyle-related characteristics including low educational attainment, smoking, adiposity, a generally unhealthy diet, and physical inactivity^5,27–31^. Most RCTs of vitamin D supplementation and fatigue have been carried out within patient groups with illnesses such as autoimmune diseases, cancers, stroke or depression. There have been few RCTs of vitamin D supplementation with self-perceived fatigue among the main outcomes in general population samples or in individuals with idiopathic fatigue. We identified a few exceptions. In an RCT of otherwise healthy individuals with fatigue and low 25OHD levels, individuals in the supplementation group (n = 58) had greater odds (OR: 2.63, 95% CI: 1.23, 5.62) of perceiving an improvement in their fatigue symptoms compared to those in the placebo group (n = 62)^18^, suggesting a clinically relevant effect. However, the confidence interval (CI) of the average raw score change on the primary fatigue outcome measure included the null in both the supplementation (−3.3, 95% CI: −14.1, 4.1) and placebo (−0.8, 95% CI: −9.0, 8.7) groups (standardized mean difference [SMD]: −0.77, 95% CI:−1.13, −0.42). Two other RCTs showed little evidence of an effect of vitamin D supplementation on fatigue symptoms in primary care patients with low 25OHD levels (intervention n = 48, placebo n = 42, SMD = −0.13, 95% CI: −0.55, 0.28)^32^ or in individuals with CFS/ME (intervention n = 21, placebo n = 24, SMD = 0.12, 95% CI: −0.47, 0.71)^21^. A large RCT of combined calcium and vitamin D supplementation in middle-aged and older women found no strong evidence of a difference in fatigue between the intervention (N = 17,101) and placebo (N = 17,056) groups (p-value = 0.764, no change score was reported)^33^. We performed a random effects meta-analysis of the three smaller RCTs reporting fatigue change scores and find that the effect of vitamin D supplementation on fatigue is inconclusive and heterogeneous (SMD: 0.29, 95% CI: −0.83, 0.24, I-squared: 77.3%, p-value for heterogeneity: 0.012).

In the absence of high-quality RCT evidence MR is an approach that can be used to strengthen causal inference in observational studies by using genetic variants associated with an environmental exposure as instrumental variables (proxies) for the exposure^34^. If vitamin D status causally affects the risk of fatigue, then genetic variants that robustly influence levels of vitamin D should be associated with fatigue to the extent predicted by their influence on vitamin D status^35^.

**Figure 1:**
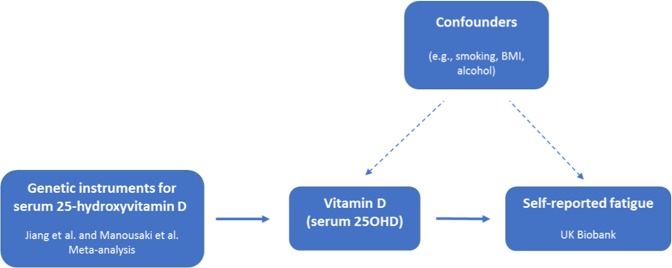
Directed acyclic graph of the Mendelian randomization study design. We selected seven independent single nucleotide polymorphisms (SNPs) robustly associated with 25-hydroxyvitamin D (25OHD) levels as genetic instruments (leftmost box) to proxy circulating levels of 25OHD. The SNPs were weighted according to the strength of their association with 25OHD in the largest available genome-wide association study (GWAS) of 25OHD (n = 42,274 for rs117913124 and n = 79,366 for the remaining six SNPs). The genetic instruments were then used to estimate if 25OHD causally influences self-reported fatigue in the independent UK Biobank sample (n = 327,478). The genetic instruments should be unrelated to potential confounders of the 25OHD-fatigue association and should only affect fatigue through 25OHD.

In the present study, we used two-sample MR to assess if genetically lowered vitamin D status causally influences fatigue in the general population (Fig. 1). As instruments for vitamin D status we used independent single nucleotide polymorphisms (SNPs) with robust evidence for association with 25OHD levels^36–38^. We then used MR methods to examine if 25OHD-lowering SNPs are associated with the risk of feeling fatigued in an independent sample of 327,478 individuals from UK Biobank.

## Methods

### Study population

UK Biobank is a population-based health research resource consisting of approximately 500,000 people, aged 38–73 years, recruited between the years 2006 and 2010 from across the UK^39^. Participants provided a range of information (such as demographics, health status, lifestyle measures, cognitive testing, personality self-report, and physical and mental health measures) via questionnaires and interviews; anthropometric measures, BP readings and samples of blood, urine and saliva were also taken (data available at http://www.ukbiobank.ac.uk). A full description of the study design, participants and quality control (QC) methods have been described in detail previously^40^. UK Biobank received ethical approval from the Research Ethics Committee (REC reference 11/NW/0382). Written informed consent was obtained from each participant. The methods were carried out in accordance with the relevant guidelines and regulations.

### Genotyping, imputation and quality control

The full data release contains the cohort of successfully genotyped samples (n = 488,377). 49,979 individuals were genotyped using the UK BiLEVE array and 438,398 using the UK Biobank axiom array. Pre-imputation QC, phasing and imputation are described elsewhere^41^. Sample QC included removing individuals of non-European ancestry, high relatedness to others in the sample, or with sex-mismatch. Details of the QC and imputation process has been described previously^42^.

### Fatigue

Fatigue was assessed in UK Biobank using the question “Over the past two weeks, how often have you felt tired or had little energy?” which was part of the Mental Health Questionnaire consisting of items from the Patient Health Questionnaire. The responses given at the initial assessment visit were used in this analysis. Participants answering with “Do not know”’ or “Prefer not to answer” were excluded, resulting in a sample of 327,478 individuals with available genotype data and fatigue measurement (“Not at all” n = 154,926, 47.31%, “Several days” 134,204, 40.98%, “More than half the days” n = 18,822, 5.75%, and “Nearly every day” n = 19,526, 5.96%). Consistent with a previously used definition of clinically significant fatigue^10^, we dichotomized the ordinal fatigue variable into fatigue experienced nearly every day (n = 19,526) or less (n = 307,952).

### SNP selection

To construct a genetic instrument for vitamin D status, we selected SNPs that were robustly (*p*-values < 5 × 10^−8^) associated with circulating 25OHD-levels in individuals of European ancestry in the largest meta-analysis to date. Four genome-wide significant SNPs were identified from the SUNLIGHT consortium^36^ (rs3755967, rs12785878, rs10741657 and rs17216707), all located in or near four 25OHD-related genes: Group-Specific Component (*GC*), Cytochrome P450 family 2, subfamily R, polypepetide 1 (*CYP2R1*), 7-Dehydrocholesterol Reductase (*DHCR7*) and Cytochrome P450, family 24, polypepetide 1 (*CYP24A1*). These have been used as genetic instruments for 25OHD in MR studies previously^43,44^ and have shown little association with potential confounders such as BMI, smoking, diet and sunlight exposure^37,43,45^. In more recent and larger meta-analyses, three additional SNPs have shown genome-wide significant association with circulating 25OHD-levels: rs117913124 in *CYP2R1*^37^, rs8018720 in *SEC23A*^38^, and rs10745742 in *AMDHD1*^38^. The seven SNPs (see Supplementary Table 1) are independent (r^2^ > 0.8) and together explain approximately 5% of the variance in circulating 25OHD levels^37,38^.

Effect estimates for the association of the SNPs with circulating 25OHD-concentrations were taken from the most recent meta-analysis^38^ apart from rs117913124, for which the effect estimate was only available from Manousaki *et al*.^37^. The beta and standard error (SE) for rs117913124 were scaled to match the other SNPs by plotting the betas of the other SNPs against each other and multiplying both the beta and SE by the beta of the regression line.

The beta values reflect changes in standard deviations (SD) of the standardized log-transformed levels of 25OHD^37^. Weighting by these beta coefficients means that each unit increase represents a standard deviation increase in log25OHD. The GWAS summary dataset and UK Biobank dataset were harmonized so that the effect allele for all seven SNPs was the 25OHD-decreasing allele^46^.

### Covariates

All analyses were adjusted for sex, genetic batch, assessment centre and all of the 40 principal components as provided by UK Biobank to correct for population structure.

### Statistical analysis

#### Mendelian randomization (MR) analysis

MR is an instrumental variable analysis based on a ratio of the regressions of the genetic instrument-outcome association (weighted 25OHD-SNPs with self-reported fatigue in UK Biobank) on the genetic instrument-exposure association (25OHD-SNPs with circulating 25OHD in the independent vitamin D GWAS). The individual SNP-fatigue estimates were pooled using three MR methods, each with different assumptions. The inverse-variance weighted (IVW) estimate is the inverse variance weighted mean of the ratio estimates obtained for all the genetic instruments^47^, here the 25OHD SNPs. The IVW method assumes that all the SNPs included are valid instruments for 25OHD-levels. The weighted median employs the weighted empirical distribution function of each SNP ratio estimate and provides the median of them. This method yields a consistent estimate of a true causal effect if more than 50% of SNPs are valid^48^.Finally, MR Egger is a weighted linear regression and provides a consistent estimate of a true causal effect even if all SNPs are invalid instruments, assuming that pleiotropic effects are independent of instrument strength^49^. In addition, the intercept provides a measure of horizontal pleiotropy, the absence of which would lead to a null y-intercept (ie the mean value of the SNP-vitamin D associations when the SNP-tiredness association is zero).The MR estimates are reported as odds ratios (OR) with their 95% confidence intervals (CI) for fatigue per genetically determined 1-SD reduction in standardized log-transformed 25OHD-levels. We estimated our statistical power using the online tool mRnd (http://cnsgenomics.com/shiny/mRnd/) (alpha = 0.05, r^2^ = 0.05). There was 85% power to detect a relative 10% difference in risk of high fatigue for 1 SD difference in log-transformed 25OHD (i.e., an OR of at least 1.10 or 0.90).

#### Sensitivity analyses

For MR to provide valid results, the alleles used to instrument the exposure must be a) robustly associated with the exposure, b) independent of confounding factors, and c) only influence the outcome through the exposure. Assumption a) was met by including only SNPs robustly associated with vitamin D status. The remaining assumptions cannot be empirically tested and potential violations were considered in sensitivity analyses.

To test the assumption that the genetic instrument is not associated with factors that typically confound the observational association, potential confounders such as sex (coded in the UK Biobank showcase as Data-Field 31), smoking status (Data-Field 20116 – coded as never, ever, current), alcohol use (Data-Field 20117 – coded as never and ever) and assessment centre (Data-Field 54), were examined for associations with the 25OHD instrument. For each individual a polygenic risk score was generated combining the seven 25OHD variants weighted by the beta coefficients mentioned above. This resulted in a score that is on the standardized log 25OHD scale. Associations of the 25OHD instrument with strata of potential confounders were tested using linear regression with categorical variables as factor variables. Boxplots display means and range +/− one standard deviation and the overall p-value for the model.

The MR assumption that is most likely to be violated is c); assuming that the genetic instrument only influences the outcome through the exposure. To detect potential horizontal pleiotropy (the SNPs influencing self-reported fatigue through other pathways than through circulating vitamin D), we assessed the intercept of the Egger regression. The Cochran’s Q statistic was also calculated to estimate whether the estimate of the causal effect of vitamin D on fatigue was inconsistent across the individual SNPs. To identify potentially influential SNPs, which could be driven for example by horizontal pleiotropy, we used leave-one-out analyses and performed single-SNP MR for each of the seven SNPs.

We examined if the association of the genetic instrument with fatigue differed across the categories of fatigue. Given that the threshold used to define high fatigue may be arbitrary, and the possibility that the association between 25OHD and fatigue varies by level of fatigue, we performed the MR analysis with the fatigue outcome dichotomized at each of the other response categories as well as as a continuous variable.

We performed a bidirectional MR investigating the effect of tiredness on 25OHD level using the significantly associated SNPs from the GWAS of the frequency of tiredness performed by the Neale group (http://www.nealelab.is/blog/2017/9/11/details-and-considerations-of-the-uk-biobank-gwas) and the vitamin D meta-analysis summary statistics^38^.

We investigated the enrichment of common disorders, typically associated with tiredness, across the categories of tiredness. These disorders were defined through ICD-10 codes (UK Biobank data-field (DF) 41202), the non-cancer coding illness questionnaire (UK Biobank DF 20002) or answers to questions. The disorders were multiple sclerosis (DF = 41202, data-coding (DC) = G35 and DF = 20002, DC = 1261), rheumatoid arthritis (DF = 41202, DC = M05, M053, M058, M059, M06, M060, M068, M069, M08, M080 and DF = 20002, DC = 1464), inflammatory bowel disease (DF = 41202, DC = K58 and DF = 20002, DC = 1154 and 1461), cancers (DF = 2453), depression (DF = 20002, DC = 1286), any long standing illness, disability or infirmity (DF = 2188) and chronic fatigue syndrome (DF = 41202, DC = G933 and DF = 20002, DC = 1482).

All analyses were performed in R (version 3.3.1) (http://www.r-project.org). The TwoSampleMR R package was used to perform MR analyses and sensitivity analyses^50^.

## Results

There was little evidence of association of the 25OHD genetic instruments, combined as a polygenic risk score (PRS), with sex, BMI, smoking status, alcohol intake or educational attainment. As there was some evidence for a difference in the 25OHD PRS across assessment centres, centre was included as a covariate in the analysis (Supplementary Fig. 1).

**Figure 2:**
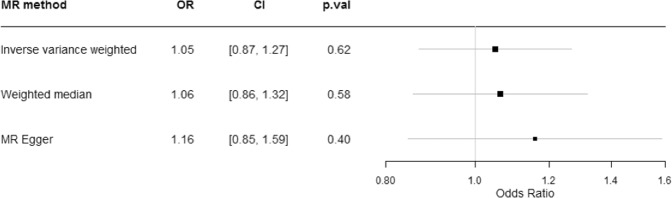
Mendelian Randomization analysis of the effect of 25OHD on fatigue. Forest plot comparing results from inverse variance weighted, weighted median and MR Egger methods. OR = odds ratio per 1 SD log unit increase in 25OHD. 25OHD = 25-hydroxyvitamin D.

**Figure 3:**
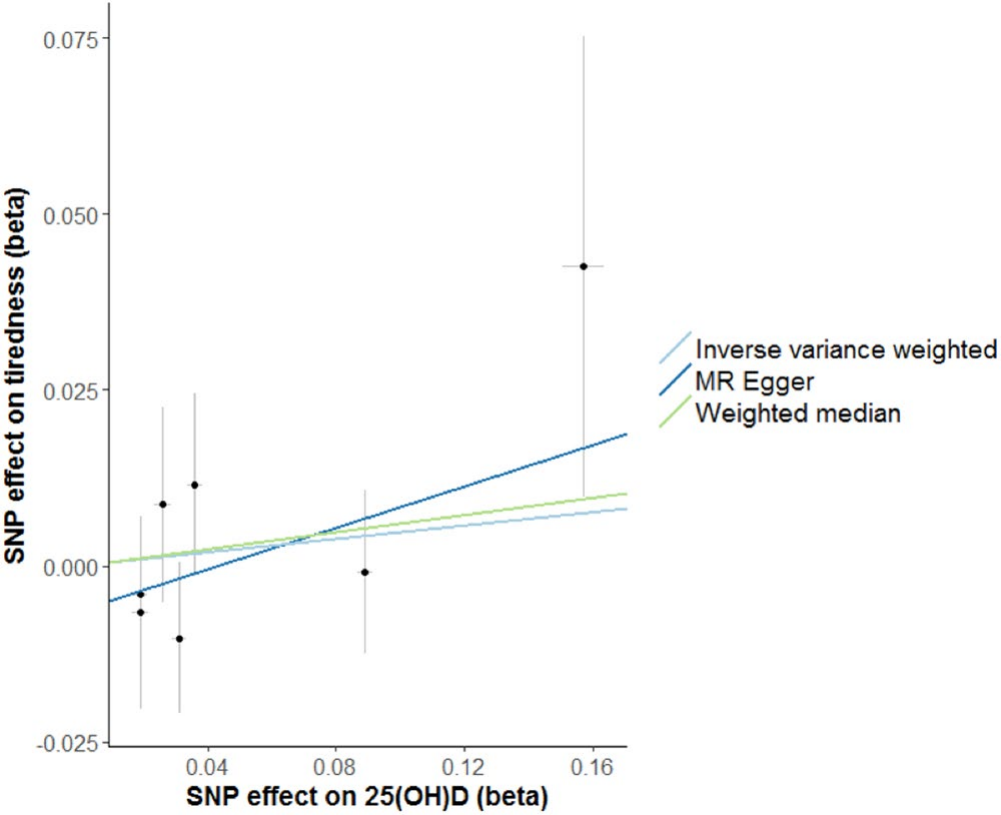
Scatter plot showing the relationship of SNP effects on 25OHD against the 25OHD SNP effects on fatigue. Each of the SNPs associated with 25OHD levels are represented by a black dot with the error bar depicting the standard error of its association with 25OHD (horizontal) and tiredness (vertical). The effects are given in betas. The slope of the lines represents the causal association as calculated using the different MR methods and provides a comparison between the three MR methods. 25OHD = 25-hydroxyvitamin D.

Consistent with a previously used definition of clinically significant fatigue^10^, we dichotomized the ordinal tiredness variable into tiredness experienced nearly every day (n = 19,526) or less (n = 307,946). We weighted the SNPs associated with 25OHD to proxy for genetically lowered 25OHD levels. The results, as displayed in Fig. 2, were consistent among the different MR methods, and showed little association between genetically lowered 25OHD levels and feeling fatigued nearly every day. Odds ratios were 1.05 (95% CI 0.87–1.27), 1.06 (95% CI, 0.86–1.32) and 1.16 (95% CI, 0.85–1.59) per 1-SD change in log 25OHD levels using IVW, weighted median and MR Egger approaches, respectively. The scatter plot in Fig. 3 depicts the relationship between the effect of the SNPs associated with vitamin D and their effects on tiredness. Given that MR is an instrumental variable analysis based on a ratio of the regressions of the weighted 25OHD-SNPs with self-reported fatigue (calculated in UK Biobank) on the 25OHD-SNPs with circulating 25OHD (taken from the independent vitamin D GWAS), the slope of the lines represents the causal association as calculated using the different MR methods and provides a comparison between the three MR methods. These show no strong evidence for a causal effect of circulating 25OHD levels on fatigue.

The small MR Egger intercept (−0.005, p = 0.25) suggests no strong unbalanced horizontal pleiotropy. In addition, the Cochran Q statistic of 3.91 with an associated *P* value of 0.69 shows no strong evidence of heterogeneity in the analysis.

**Figure 4:**
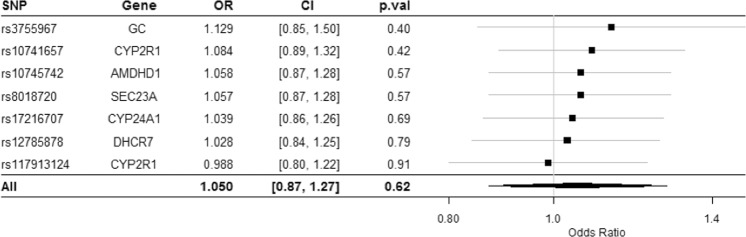
Leave-one-out sensitivity analysis of the MR analysis (using IVW) excluding that particular SNP. IVW = inverse-variance weighted method.

In the leave-one-out analysis, sequentially omitting each of the seven SNPs, all OR estimates of fatigue per 1-SD change in circulating 25OHD levels were similar and all crossed the null (Fig. 4).

There was little evidence that the 25OHD genetic instrument is associated with any level of fatigue (Supplementary Fig. 2), and the MR estimates were similar when using alternative thresholds to define fatigue (Supplementary Fig. 3–5) or using a continuous variable (Supplementary Fig. 6). The effect of fatigue on vitamin D status was assessed and showed little association between genetically lowered fatigue levels and 25OHD (Supplementary Fig. 7).

We expanded this study to investigate the enrichment of common disorders across the categories of tiredness (Supplementary Fig. 8). The proportion of individuals who responded that they are tired “nearly every day” was not greater than those responding that they are “not at all” tired (except for chronic fatigue syndrome).

## Discussion

This is the first study to date using MR to test if the association between vitamin D status and fatigue is likely to be causal. We found little evidence of a causal association using a large population of individuals with European descent. These findings suggest that vitamin D supplementation might not be helpful in reducing the risk of feeling fatigued.

It is possible that previously reported observational associations reflect unmeasured or residual confounding from factors influencing both vitamin D status and fatigue. For example, adiposity may influence fatigue^28,51^ and reduce vitamin D status^44^. Reverse causation could also explain the discrepancy between our MR results and observational studies. Given that individuals feeling fatigued tend to be less physically active and to stay indoors^27,28^, they receive less exposure to sunlight. The MR approach reduces the risk of confounding, as genetic variants are much less associated with socioeconomic and lifestyle factors than directly measured environmental exposures (e.g., 25OHD)^52,53^. The MR results should be less vulnerable to both confounding and reverse causation given the random allocation of genetic variants at meiosis and that germline genotypes do not change thereafter.

To the best of our knowledge, only four vitamin D RCTs have been conducted with self-perceived fatigue as one of the main outcomes in general population samples or individuals with idiopathic fatigue. Our results are in line with the RCTs showing little evidence of improvement in fatigue after eight weeks of vitamin D supplementation in primary care patients with low 25OHD levels (N = 90; SMD = −0.13, 95% CI: −0.55, 0.28)^32^, after six months of vitamin D supplementation in patients with chronic fatigue syndrome (N = 45; SMD = 0.12, 95% CI: −0.47, 0.71)^21^, or over an average of five years of combined calcium and vitamin D supplementation in middle-aged and older women (N = 34,157; p = 0.764)^33^.

However, the results of our study are not in accordance with Nowak *et al*.^18^, a trial (N = 120) reporting OR of 2.63 (95% CI: 1.23, 5.62) for self-perceived amelioration of fatigue four weeks after a single high-dose of vitamin D supplementation. The treatment group in Nowak *et al*. received 100,000 IU vitamin D (cholecalciferol) and showed a mean increase in 25OHD levels by 35 nmol/L (14 μg/L) from a mean baseline level of 33 nmol/L (13 μg/L). A previous MR study of vitamin D status reported that a 1-SD increase in log-transformed levels of 25OHD was associated with an increase in circulating 25OHD levels comparable in effect size to that obtained through vitamin D supplementation^54^. The effect of 1-SD increase in log-transformed 25OHD levels on circulating 25OHD was 35.6 nmol/L in vitamin D sufficient individuals (50–75 nmol/L), 23.7 nmol/L in vitamin D insufficient individuals (25–49 nmol/L), and 11.9 nmol/L in vitamin D deficient individuals (<25 nmol/L)^54^. Provided that this assumption holds here, there was power to exclude an effect of the size reported by Nowak *et al*. This RCT had a short follow-up period compared with the RCTs with null results. An important difference between RCTs and our MR study design is that RCTs assess the short-term effects of vitamin D supplementation on fatigue, whereas MR methods assess the association of a lifelong exposure to reduced vitamin D status with fatigue^35^. We cannot exclude that vitamin D supplementation may influence fatigue in individuals who have had a sudden reduction from their lifelong vitamin D status.

The main strengths of this study are the large sample size of the UK Biobank, the robust genetic instruments developed from a GWAS meta-analysis explaining approximately 5% of the variance in 25OHD levels (the explained phenotypic variance in MR studies is often around 1%)^55^, and the use of multiple MR methods with different assumptions. Given that we relied on a single question to define fatigue (“Over the past two weeks, how often have you felt tired or had little energy?”), there may be some outcome misclassification. Thus, additional research is needed to assess if our results replicate when a more comprehensive measure of fatigue is used such as the Patient Reported Outcomes Measurement Information System (PROMIS) fatigue item bank^56^. The restriction of the analyses to white populations of European ancestry was necessary to avoid confounding by population stratification, but also limits interpretation of the results to this population.

We explored potential pleiotropy by using MR methods designed to account for pleiotropic effects and assessed associations between the vitamin D-reducing SNPs and putative confounders. However, the possibility of pleiotropic SNP effects cannot be excluded. Our understanding of the proximal functional consequences of the SNPs (beyond the association with 25OHD) is limited. In addition, the genetic instrument specifically proxies average total (free and bound) circulating 25OHD and does not necessarily predict the concentrations of free 25OHD available at tissues or concentrations of free or bound 1,25OHD_2_D in circulation or at the tissue level. Therefore we restrict our interpretation of these results to circulating 25OHD. Moreover, there is a possibility that an effect of 25OHD on fatigue may be obscured by compensatory feedback mechanisms (canalization)^57^. This is most likely to occur in the early (generally prenatal) stage of the life course. The present study was underpowered to exclude a small causal effect (<0.90 OR >1.10), and the confidence intervals of some of the MR estimates included potentially non-negligible effects. A limitation of the MR design is that we cannot rule out the possibility that individuals with frank vitamin D deficiency may benefit from vitamin D supplementation.

In conclusion, the results of our MR analyses provide no strong evidence of a causal association between vitamin D status and self-reported fatigue. Our findings suggest that efforts to increase vitamin D status in the general population are unlikely to have a clinically relevant influence on fatigue symptoms.

## Supplementary information

Supplementary information

## Data Availability

The data reported in this paper are available via application directly to the UK Biobank (http://www.ukbiobank.ac.uk/). The summary statistics on the 25OHD-associated SNPs are included in the Supplementary Information.

## References

[1] Meng H, Hale L, Friedberg F (2010). Prevalence and predictors of fatigue among middle-aged and older adults: Evidence from the health and retirement study. J. Am. Geriatr. Soc..

[2] Pawlikowska T (1994). Population based study of fatigue and psychological distress. BMJ.

[3] Ricci JA, Chee E, Lorandeau AL, Berger J (2007). Fatigue in the U.S. workforce: Prevalence and implications for lost productive work time. J. Occup. Environ. Med..

[4] van’t Leven M, Zielhuis GA, van der Meer JW, Verbeek AL, Bleijenberg G (2010). Fatigue and chronic fatigue syndrome-like complaints in the general population. Eur. J. Public Health.

[5] Autier P, Boniol M, Pizot C, Mullie P (2014). Vitamin D status and ill health: A systematic review. Lancet Diabetes Endocrinol..

[6] Fukuda K (1994). The chronic fatigue syndrome: A comprehensive approach to its definition and study. Ann. Intern. Med..

[7] Brurberg KG, Fønhus MS, Larun L, Flottorp S, Malterud K (2014). Case definitions for chronic fatigue syndrome/myalgic encephalomyelitis (CFS/ME): A systematic review. BMJ Open.

[8] Cathébras PJ, Robbins JM, Kirmayer LJ, Hayton BC (1992). Fatigue in primary care. J. Gen. Intern. Med..

[9] David A (1990). Tired, weak, or in need of rest: Fatigue among general practice attenders. BMJ.

[10] Lynch J (2007). Fatigue after stroke: The development and evaluation of a case definition. J. Psychosom. Res..

[11] Walker EA, Katon WJ, Jemelka RP (1993). Psychiatric disorders and medical care utilization among people in the general population who report fatigue. J. Gen. Intern. Med..

[12] Askmark H, Haggård L, Nygren I, Punga AR (2012). Vitamin D deficiency in patients with myasthenia gravis and improvement of fatigue after supplementation of vitamin D3: A pilot study. Eur. J. Neurol..

[13] Ruiz-Irastorza G, Egurbide MV, Olivares N, Martinez-Berriotxoa A, Aguirre C (2008). Vitamin D deficiency in systemic lupus erythematosus: Prevalence, predictors and clinical consequences. Rheumatology.

[14] Raftery T (2013). Supplemental vitamin D in quiescent Crohn’s disease–effects on quality of life, fatigue and muscle strength: Results from a double blind placebo controlled study. Proc. Nutr. Soc..

[15] Roy S, Sherman A, Monari-Sparks MJ, Schweiker O, Hunter K (2014). Correction of low vitamin D improves fatigue: Effect of correction of low vitamin D in fatigue study (EViDiF Study). N. Am. J. Med. Sci..

[16] Martínez-Alonso M, Dusso A, Ariza G, Nabal M (2015). Vitamin D deficiency and its association with fatigue and quality of life in advanced cancer patients under palliative care: A cross-sectional study. Palliat. Med..

[17] Lima GL (2016). Vitamin D supplementation in adolescents and young adults with juvenile systemic lupus erythematosus for improvement in disease activity and fatigue scores: A randomized, double-blind, placebo-controlled trial. Arthritis Care Res..

[18] Nowak A (2016). Effect of vitamin D3 on self-perceived fatigue: A double-blind randomized placebo-controlled trial. Medicine (Baltimore).

[19] Frigstad SO (2017). Fatigue is not associated with vitamin D deficiency in IBD patients. Gastroenterology.

[20] Knippenberg S (2014). Higher levels of reported sun exposure, and not vitamin D status, are associated with less depressive symptoms and fatigue in multiple sclerosis. Acta Neurol. Scand..

[21] Witham MD (2015). Effect of intermittent vitamin D3 on vascular function and symptoms in chronic fatigue syndrome – A randomised controlled trial. Nutr. Metab. Cardiovasc. Dis..

[22] Berkovitz S, Ambler G, Jenkins M, Thurgood S (2009). Serum 25-hydroxy vitamin D levels in chronic fatigue syndrome: A retrospective survey. Int. J. Vitam. Nutr. Res..

[23] Spritzler, F. 8 Signs and Symptoms of Vitamin D Deficiency. *Healthline*, http://www.healthline.com/nutrition/vitamin-d-deficiency-symptoms (2016).

[24] Ostler, C. Is this the surprise reason you’re always tired? How vitamin D can give you more energy and lower blood pressure. *Daily Mail Online*, http://www.dailymail.co.uk/femail/article-3404027/Is-surprise-reason-tired-vitamin-D-energy-lower-blood-pressure.html (2016).

[25] Tapley A (2015). Test ordering in an evidence free zone: Rates and associations of Australian general practice trainees’ vitamin D test ordering. J. Eval. Clin. Pract..

[26] Woodford HJ, Barrett S, Pattman S (2018). Vitamin D: too much testing and treating?. Clin. Med..

[27] Engberg I, Segerstedt J, Waller G, Wennberg P, Eliasson M (2017). Fatigue in the general population- Associations to age, sex, socioeconomic status, physical activity, sitting time and self-rated health: the northern Sweden MONICA study 2014. BMC Public Health.

[28] Tønnesen R, Hovind PH, Jensen LT, Schwarz P (2016). Determinants of vitamin D status in young adults: influence of lifestyle, sociodemographic and anthropometric factors. BMC Public Health.

[29] Theorell-Haglow J, Lindberg E, Janson C (2006). What are the important risk factors for daytime sleepiness and fatigue in women?. Sleep.

[30] Ginde AA, Liu MC, Camargo CA (2009). Demographic differences and trends of vitamin D insufficiency in the US population, 1988–2004. Arch. Intern. Med..

[31] Goedendorp MM, Knoop H, Schippers GM, Bleijenberg G (2009). The lifestyle of patients with chronic fatigue syndrome and the effect on fatigue and functional impairments. J. Hum. Nutr. Diet.

[32] Arvold DS (2009). Correlation of symptoms with vitamin D deficiency and symptom response to cholecalciferol treatment: a randomized controlled trial. Endocr. Pract..

[33] LeBlanc ES (2015). Calcium and vitamin D supplementation do not influence menopause-related symptoms: Results of the Women’s Health Initiative Trial. Maturitas.

[34] Davey Smith G, Ebrahim S (2003). ‘Mendelian randomization’: Can genetic epidemiology contribute to understanding environmental determinants of disease?. Int. J. Epidemiol..

[35] Davey Smith G (2011). Use of genetic markers and gene-diet interactions for interrogating population-level causal influences of diet on health. Genes. Nutr..

[36] Wang TJ (2010). Common genetic determinants of vitamin D insufficiency: a genome-wide association study. Lancet.

[37] Manousaki D (2017). Low-frequency synonymous coding variation in CYP2R1 has large effects on vitamin D levels and risk of multiple sclerosis. Am. J. Hum. Genet..

[38] Jiang X (2018). Genome-wide association study in 79,366 European-ancestry individuals informs the genetic architecture of 25-hydroxyvitamin D levels. Nat. Commun..

[39] Allen NE, Sudlow C, Peakman T, Collins RUK (2014). Biobank data: Come and get it. Sci Transl Med.

[40] Collins R (2012). What makes UK Biobank special?. Lancet.

[41] Bycroft, C. *et al*. Genome-wide genetic data on ~500,000 UK Biobank participants. *BioRxiv*, Preprint at, 10.1101/166298 (2017).

[42] Mitchell, R., Hemani, G., Dudding, T. & Paternoster, L. UK Biobank Genetic Data: MRC-IEU Quality Control, Version 1, 10.5523/bris.3074krb5526t5522frj5529yh5522b5503x5523wxj (2017).

[43] Mokry LE (2015). Vitamin D and Risk of Multiple Sclerosis: A Mendelian Randomization Study. PLoS Med..

[44] Vimaleswaran KS (2013). Causal relationship between obesity and vitamin D status: Bi-directional Mendelian randomization analysis of multiple cohorts. PLoS Med..

[45] Berry DJ, Vimaleswaran KS, Whittaker JC, Hingorani AD, Hyppönen E (2012). Evaluation of genetic markers as Instruments for Mendelian randomization studies on vitamin D. PLoS ONE.

[46] Hartwig FP, Davies NM, Hemani G, Davey Smith G (2016). Two-sample Mendelian randomization: Avoiding the downsides of a powerful, widely applicable but potentially fallible technique. Int. J. Epidemiol..

[47] Burgess S, Butterworth A, Thompson SG (2013). Mendelian Randomization Analysis With Multiple Genetic Variants Using Summarized Data. Genet. Epidemiol..

[48] Hemani G, Bowden J, Davey Smith G (2018). Evaluating the potential role of pleiotropy in Mendelian randomization studies. Hum. Mol. Genet..

[49] Burgess S, Bowden J, Fall T, Ingelsson E, Thompson SG (2017). Sensitivity analyses for robust causal inference from mendelian randomization analyses with multiple genetic variants. Epidemiology.

[50] Hemani, G. *et al*. MR-Base: A platform for systematic causal inference across the phenome using billions of genetic associations. *BioRxiv*, Preprint at, 10.1101/078972 (2016).

[51] Norris, T., Hawton, K., Hamilton-Shield, J. & Crawley, E. Obesity in adolescents with chronic fatigue syndrome: an observational study. *Arch. Dis. Child* (2016). 10.1136/archdischild-2016-311293 PMC5256402 27655658

[52] Taylor, M. *et al*. Testing the principles of Mendelian randomization: Opportunities and complications on a genomewide scale. *BioRxiv*, Preprint at, 10.1101/124362 (2017).

[53] Davey Smith G (2007). Clustered environments and randomized Genes: A fundamental distinction between conventional and genetic epidemiology. PLoS Med..

[54] Manousaki, D., Mokry, L. E., Ross, S., Goltzman, D. & Richards, J. B. Mendelian randomization studies do not support a role for vitamin D in coronary artery disease. *Circ. Cardiovasc. Genet* (2016). 10.1161/CIRCGENETICS.116.001396 27418593

[55] Pierce BL, Ahsan H, VanderWeele TJ (2011). Power and instrument strength requirements for Mendelian randomization studies using multiple genetic variants. Int. J. Epidemiol..

[56] Lai J-S (2011). How item banks and their application can influence measurement practice in rehabilitation medicine: A PROMIS fatigue item bank example. Arch. Phys. Med. Rehabil..

[57] Lawlor DA, Harbord RM, Sterne JAC, Timpson N, Davey Smith G (2008). Mendelian randomization: Using genes as instruments for making causal inferences in epidemiology. Stat. Med..

