# Author Correction: Investigating causality in the association between vitamin D status and self-reported tiredness

**DOI:** 10.1038/s41598-021-89274-5

**Published:** 2021-05-07

**Authors:** Alexandra Havdahl, Ruth Mitchell, Lavinia Paternoster, George Davey Smith

**Affiliations:** 1 Medical Research Council Integrative Epidemiology Unit, University of Bristol, Bristol, BS8 2BN United Kingdom; 2 Population Health Sciences, Bristol Medical School, University of Bristol, Bristol, BS8 2BN United Kingdom; 3 Nic Waals Institute, Lovisenberg Diaconal Hospital, 0853 Oslo, Norway; 4 Department of Mental Disorders, Norwegian Institute of Public Health, N-0213 Oslo, Norway; 5 National Institute for Health Research Bristol Biomedical Research Centre, University Hospitals Bristol NHS Foundation Trust and University of Bristol, Bristol, UK

Correction to: *Scientific Reports* https://doi.org/10.1038/s41598-019-39359-z, published online 27 February 2019

This Article contains errors.

The weights used in the Mendelian Randomisation (MR) framework presented in the manuscript are natural-log transformed 25-hydroxyvitamin D, not per one standard deviation change in the log 25-hydroxyvitamin D levels as previously reported.

Additionally, the authors report MR estimates per standard deviation change in log transformed 25-hydroxyvitamin D, which were previously omitted.

As a result, in the Abstract,

“Using seven genome-wide significant 25OHD-reducing genetic variants, there was little evidence for a causal effect of 25OHD on fatigue (odds ratio for fatigue was 1.05 with 95% confidence interval of 0.87–1.27 per 1-SD decrease in log-transformed 25OHD).”

now reads:

“Using seven genome-wide significant 25OHD-reducing genetic variants, there was little evidence for a causal effect of 25OHD on fatigue [odds ratio for fatigue was 1.05 with 95% confidence interval (CI) of 0.87–1.27 per unit decrease in log-transformed 25OHD (1.02 with 95% CI of 0.99–1.06 per 1-SD decrease in log-transformed 25OHD)].”

In the Methods section, subheading “SNP selection”,

“The beta values reflect changes in standard deviations (SD) of the standardized log-transformed levels of 25OHD^37^. Weighting by these beta coefficients means that each unit increase represents a standard deviation increase in log25OHD”.

now reads:

“The beta values reflect changes in natural log-transformed concentrations of 25OHD^38^. Weighting by these beta coefficients means that each unit change represents a unit change in log25OHD levels. To ease interpretation, we also report a change in standard deviations (SD) of log-transformed levels of 25OHD by weighting with betas from an independent GWAS reporting beta coefficients for the association between SNPs and standardized log-transformed levels of 25OHD^37^."

Under subheading “Statistical analysis”, “Mendelian randomization (MR analysis)”,

“The MR estimates are reported as odds ratios (OR) with their 95% confidence intervals (CI) for fatigue per genetically determined 1-SD reduction in standardized log-transformed 25OHD-levels.”

now reads:

“The MR estimates are reported as odds ratios (OR) with their 95% confidence intervals (CI) for fatigue per genetically determined unit reduction in log-transformed 25OHD-levels”.

In the Results section,

“Odds ratios were 1.05 (95% CI 0.87–1.27), 1.06 (95% CI, 0.86–1.32) and 1.16 (95% CI, 0.85–1.59) per 1-SD change in log 25OHD levels using IVW, weighted median and MR Egger approaches, respectively. “

now reads:

“Odds ratios were 1.05 (95% CI 0.87–1.27), 1.06 (95% CI, 0.86–1.32) and 1.16 (95% CI, 0.85–1.59) per unit change in log 25OHD levels using IVW, weighted median and MR Egger approaches, respectively. Per 1-SD change in log 25OHD levels using an alternative instrument, the odds ratios were 1.02 (95% CI 0.99–1.06), 1.03 (95% CI, 0.99–1.07) and 1.05 (95% CI, 1.00–1.11), respectively.

Under the same heading,

“In the leave-one-out analysis, sequentially omitting each of the seven SNPs, all OR estimates of fatigue per 1-SD change in circulating 25OHD levels were similar and all crossed the null (Fig. 4).”

now reads:

“In the leave-one-out analysis, sequentially omitting each of the seven SNPs, all OR estimates of fatigue per unit change in circulating 25OHD levels were similar and all crossed the null (Fig. 4).”

In the legend of Fig. 2,

“OR = odds ratio per 1 SD log unit increase in 25OHD. 25OHD = 25-hydroxyvitamin D.”

now reads:

“OR = odds ratio per unit decrease in 25OHD.”

As a result of the changes, the Supplementary Information file is incorrect.

In Supplementary Figure legends S3, S4 and S5,

“OR = odds ratio per 1SD log unit increase in 25OHD” now reads: “OR = odds ratio per unit decrease in log-25OHD.”

In Supplementary Figure legends S6 and S7,

“Beta = Increase fatigue category per 1SD log unit increase in 250HD” now reads: “Beta – Increase fatigue category per unit decrease in log-250HD”.

Furthermore, a Supplementary Table showing “characteristics of SNPs associated with 25-hydroxyvitatmin D used as instrumental variables reporting odd ratios per 1 standard deviation change in log-transformed 25-OHD” was previously omitted. The correct table now appears as Supplementary Table 2.

The original Supplementary Information is linked to this Correction notice.

The original Article and accompanying Supplementary Information file has been corrected.

## Supplementary Information

Supplementary Information

