# Supplementary material for: Investigating causality in the association between vitamin D status and self-reported tiredness
Source: Sci Rep. 2019 Feb 27;9:2880. doi: 10.1038/s41598-019-39359-z (PMC6393455; doi:10.1038/s41598-019-39359-z)
Supplement: Supplementary file 1 — Supplementary information [file 41598_2019_39359_MOESM1_ESM.pdf]

Supplementary Information

Investigating causality in the association between vitamin D status and self-reported tiredness

Alexandra Havdahl+[1,2,3,4], Ruth Mitchell+[1,2], Lavinia Paternoster[1,2], George Davey Smith[1,2,5]

[1] Medical Research Council Integrative Epidemiology Unit, University of Bristol, Bristol, BS8 2BN, United Kingdom

[2] Population Health Sciences, Bristol Medical School, University of Bristol, Bristol, BS8 2BN, United Kingdom

[3] Nic Waals Institute, Lovisenberg Diaconal Hospital, Oslo, 0853, Norway

[4] Department of Mental Disorders, Norwegian Institute of Public Health, Oslo, N-0213, Norway

[5] National Institute for Health Research Bristol Biomedical Research Centre, University Hospitals Bristol NHS Foundation Trust and University of Bristol

+Alphabetical order, these authors contributed equally to this work

* alexandra.havdahl@bristol.ac.uk

Supplementary table 1:Characteristics of SNPs associated with 25-hydroxyvitamin D used as instrumental variables. The betas, standard errors (SE) and p values denote the association between the SNP and 25-hydroxyvitamin D. The effect allele frequency (EAF) refers to the A1 allele.

SNP	Chr	Position	Gene	Beta	SE	A1	A2	EAF	p value
rs3755967	4	72828262	GC	−0.089	0.0023	T	C	0.28	4.74E–343
rs12785878	11	70845097	DHCR7	-0.036	0.0022	G	T	0.25	3.80E–62
rs10741657	11	14871454	CYP2R1	-0.031	0.0022	G	A	0.60	2.05E–46
rs17216707	20	52165769	CYP24A1	-0.026	0.0027	C	T	0.21	8.14E–23
rs117913124	11	14900931	CYP2R1	-0.157	0.0065	A	G	0.03	2.29E-88
rs10745742	12	94882660	AMDHD1	-0.019	0.002	C	T	0.61	2.10E–20
rs8018720	14	38625936	SEC23A	-0.019	0.0027	C	G	0.82	1.11E–11

Supplementary figure 1: Mean (+/- standard deviation) and range of 25-hydroxyvitamin D weighted genetic instrument across strata of potential confounders. Overall mean 25-hydroxyvitamin D weighted genetic instrument for those included in the plot indicated by dashed line. 25OHD = 25-hydroxyvitamin D, p=p value for linear model.

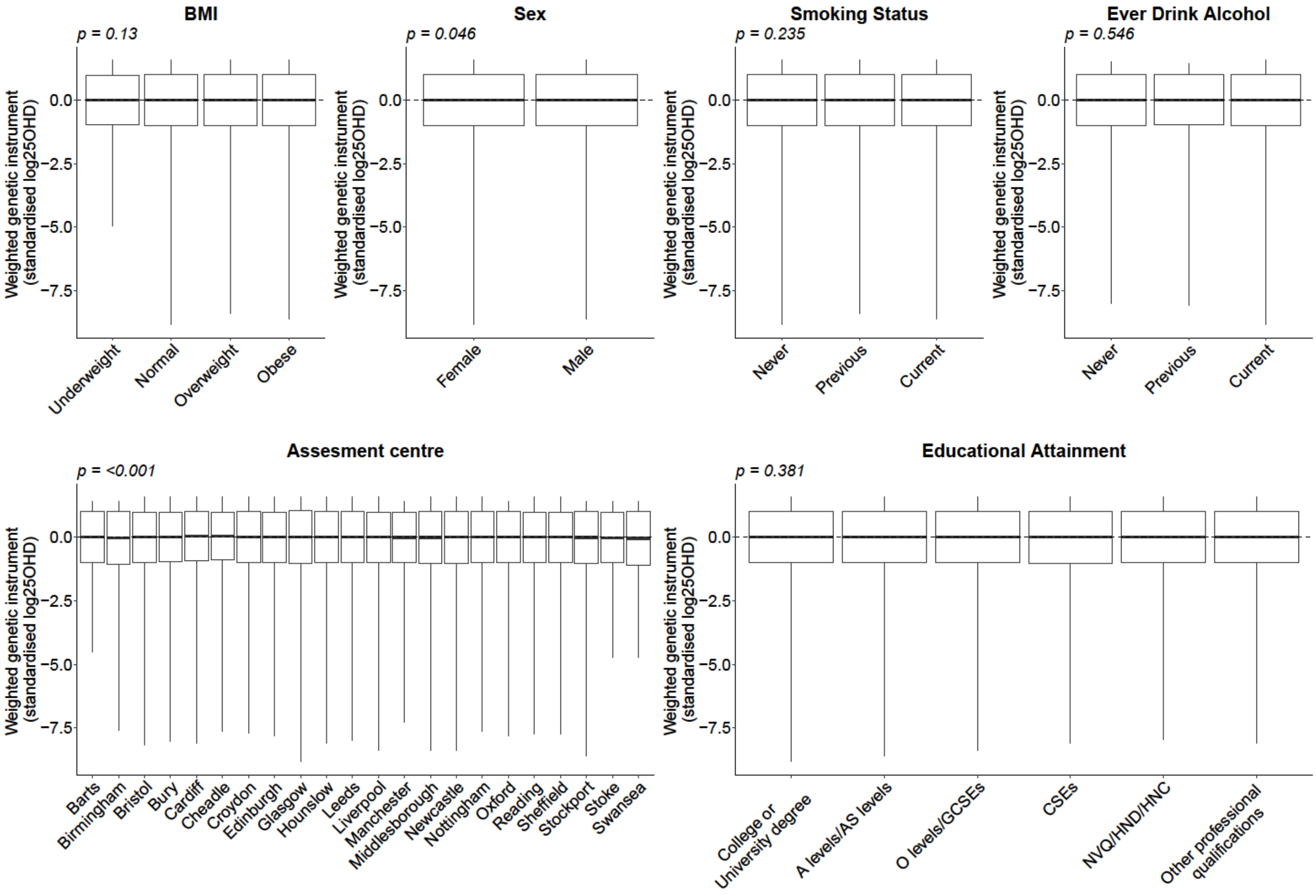

Supplementary figure 2: Mean (+/- standard deviation) and range of 25-hydroxyvitamin D weighted genetic instrument across strata of tiredness phenotype. Overall mean 25-hydroxyvitamin D weighted genetic instrument for those included in the plot indicated by dashed line. 25OHD = 25-hydroxyvitamin D.

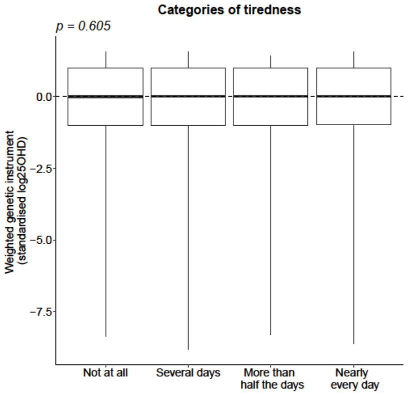

Supplementary figure 3: Mendelian Randomization analysis of the effect of 25OHD on fatigue defined as tiredness or low energy for more than half the days over the past two weeks. Forest plot comparing results from inverse variance weighted, weighted median and MR Egger methods. Leave-one-out sensitivity analysis of the MR analysis (using IVW) excluding that particular SNP. OR = odds ratio per 1SD log unit increase in 25OHD. 25OHD = 25-hydroxyvitamin D.

MR method	OR	CI	p.val
Inverse variance weighted	1.04	[0.91, 1.20]	0.55
Weighted median	1.09	[0.93, 1.28]	0.29
MR Egger	1.24	[0.98, 1.56]	0.13

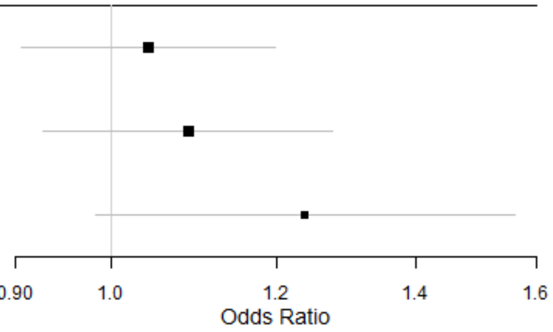

SNP	Gene	OR	CI	p.val
rs17216707	CYP24A1	1.068	[0.93, 1.23]	0.36
rs10741657	CYP2R1	1.055	[0.91, 1.22]	0.48
rs8018720	SEC23A	1.054	[0.92, 1.21]	0.46
rs10745742	AMDHD1	1.048	[0.90, 1.21]	0.53
rs12785878	DHCR7	1.041	[0.89, 1.21]	0.61
rs117913124	CYP2R1	1.014	[0.87, 1.19]	0.86
rs3755967	GC	0.988	[0.80, 1.22]	0.91
**All**		**1.043**	**[0.91, 1.20]**	**0.55**

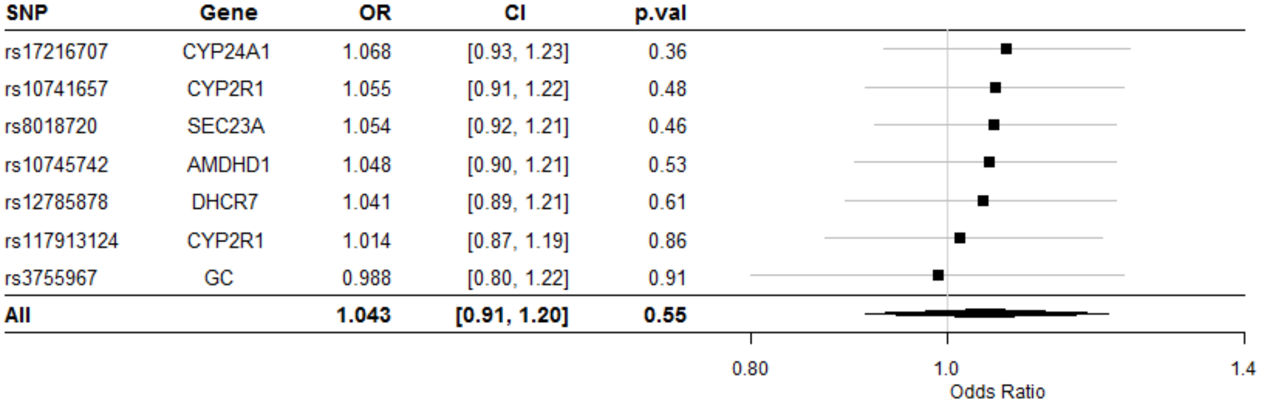

Supplementary figure 4: Mendelian Randomization analysis of the effect of 25OHD on fatigue defined as tiredness or low energy on several days or more over the past two weeks. Forest plot comparing results from inverse variance weighted, weighted median and MR Egger methods. Leave-one-out sensitivity analysis of the MR analysis (using IVW) excluding that particular SNP. OR = odds ratio per 1SD log unit increase in 25OHD. 25OHD = 25-hydroxyvitamin D.

**MR method**	**OR**	**CI**	**p.val**
Inverse variance weighted	1.06	[0.97, 1.16]	0.18
Weighted median	1.09	[0.98, 1.21]	0.11
MR Egger	1.15	[0.99, 1.34]	0.12

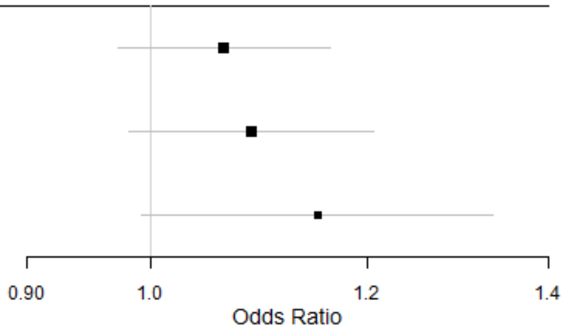

**SNP**	**Gene**	**OR**	**CI**	**p.val**
rs12785878	DHCR7	1.08	[0.98, 1.18]	0.13
rs10741657	CYP2R1	1.07	[0.98, 1.18]	0.14
rs10745742	AMDHD1	1.07	[0.98, 1.17]	0.15
rs8018720	SEC23A	1.07	[0.98, 1.17]	0.13
rs117913124	CYP2R1	1.06	[0.96, 1.18]	0.24
rs17216707	CYP24A1	1.06	[0.96, 1.16]	0.25
rs3755967	GC	1.01	[0.88, 1.15]	0.93
**All**		**1.06**	**[0.97, 1.16]**	**0.18**

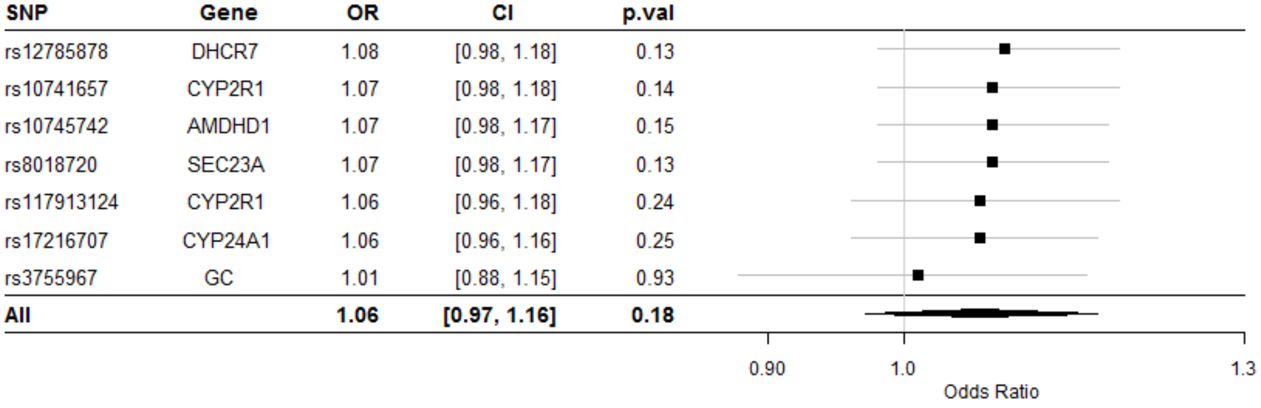

Supplementary figure 5: Mendelian Randomization analysis of the effect of 25OHD on fatigue defined as Nearly every day compared with Not at all. 25OHD = 25-hydroxyvitamin D. Forest plot comparing results from inverse variance weighted, weighted median and MR Egger methods. Leave-one-out sensitivity analysis of the MR analysis (using IVW) excluding that particular SNP. OR = odds ratio per 1SD log unit increase in 25OHD. 25OHD = 25-hydroxyvitamin D.

MR method	OR	CI	p.val
Inverse variance weighted	1.08	[0.89, 1.31]	0.45
Weighted median	1.11	[0.89, 1.39]	0.36
MR Egger	1.24	[0.90, 1.72]	0.25

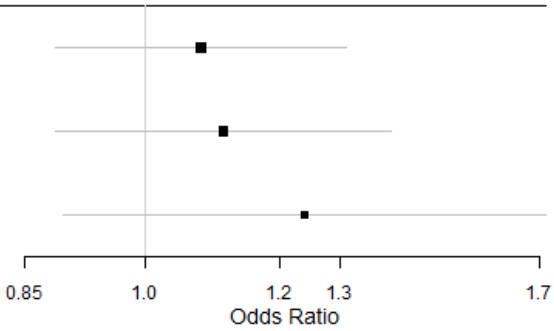

SNP	Gene	OR	CI	p.val
rs10741657	CYP2R1	1.12	[0.91, 1.37]	0.27
rs3755967	GC	1.12	[0.83, 1.5]	0.46
rs10745742	AMDHD1	1.09	[0.89, 1.33]	0.39
rs8018720	SEC23A	1.09	[0.9, 1.33]	0.38
rs12785878	DHCR7	1.06	[0.86, 1.3]	0.58
rs17216707	CYP24A1	1.06	[0.87, 1.3]	0.54
rs117913124	CYP2R1	1.02	[0.82, 1.27]	0.85
**All**		**1.08**	**[0.89, 1.31]**	**0.45**

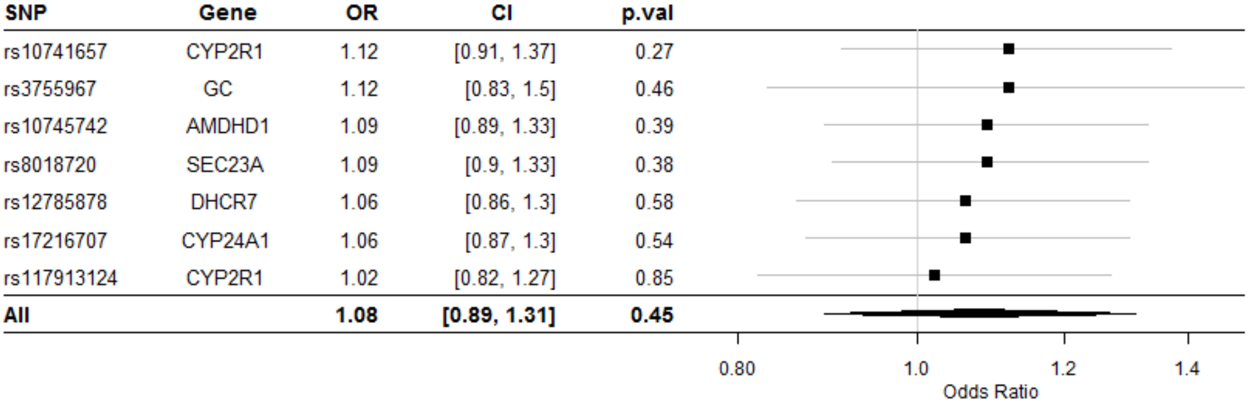

Supplementary figure 6: Mendelian Randomization analysis of the effect of 25OHD on fatigue as a continuous variable. 25OHD = 25-hydroxyvitamin D. Forest plot comparing results from inverse variance weighted, weighted median and MR Egger methods. Leave-one-out sensitivity analysis of the MR analysis (using IVW) excluding that particular SNP. Beta = Increase fatigue category per 1SD log unit increase in 25OHD. 25OHD = 25-hydroxyvitamin D.

MR method	Beta	CI	p.val
Inverse variance weighted	0.02	[-0.014, 0.059]	0.23
Weighted median	0.03	[-0.007, 0.075]	0.10
MR Egger	0.06	[0.004, 0.125]	0.09

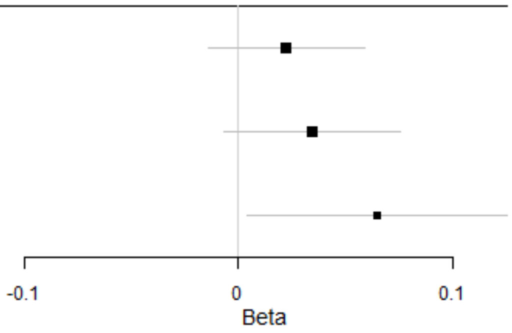

SNP	Gene	Beta	CI	p.val
rs10741657	CYP2R1	0.028	[-0.01, 0.07]	0.16
rs8018720	SEC23A	0.026	[-0.01, 0.06]	0.17
rs10745742	AMDHD1	0.024	[-0.01, 0.06]	0.20
rs12785878	DHCR7	0.024	[-0.01, 0.06]	0.22
rs17216707	CYP24A1	0.022	[-0.01, 0.06]	0.24
rs117913124	CYP2R1	0.016	[-0.03, 0.06]	0.45
rs3755967	GC	0.007	[-0.05, 0.06]	0.80
**All**		**0.022**	**[-0.01, 0.06]**	**0.23**

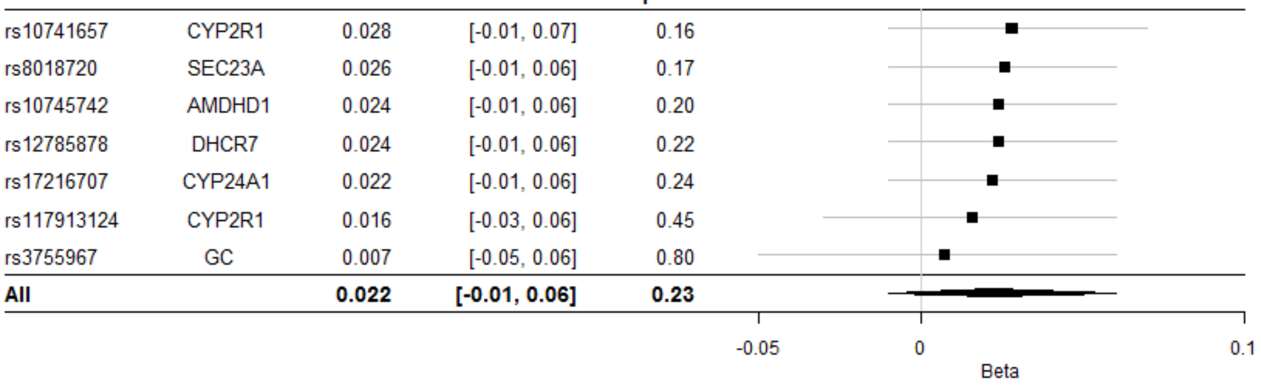

Supplementary figure 7: Mendelian Randomization analysis of the effect of tiredness on 25-hydroxyvitamin D. Forest plot comparing results from inverse variance weighted, weighted median and MR Egger methods. Leave-one-out sensitivity analysis of the MR analysis (using IVW) excluding that particular SNP. Beta= log-25OHD per 1 unit increase in fatigue. 25OHD = 25-hydroxyvitamin D.

MR method	Beta	CI	p.val
Inverse variance weighted	-0.06	[-0.17, 0.05]	0.31
Weighted median	-0.02	[-0.15, 0.11]	0.77
MR Egger	0.06	[-0.37, 0.50]	0.78

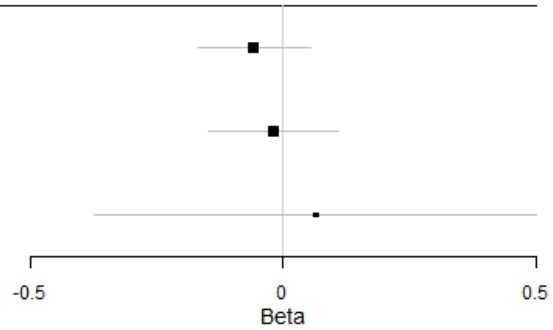

SNP	Beta	CI	p.val
rs12607755	-0.08	[-0.19, 0.03]	0.16
rs209139	-0.08	[-0.19, 0.04]	0.20
rs7036984	-0.08	[-0.19, 0.04]	0.19
rs2222765	-0.07	[-0.19, 0.04]	0.22
rs4937651	-0.07	[-0.18, 0.05]	0.25
rs7231178	-0.07	[-0.19, 0.05]	0.26
rs13139257	-0.06	[-0.18, 0.06]	0.35
rs6446257	-0.06	[-0.18, 0.06]	0.29
rs6606711	-0.06	[-0.18, 0.06]	0.32
rs2216309	-0.05	[-0.17, 0.07]	0.38
rs247975	-0.05	[-0.17, 0.07]	0.40
rs6896622	-0.05	[-0.16, 0.07]	0.44
rs7216704	-0.05	[-0.17, 0.06]	0.36
rs4690073	-0.04	[-0.14, 0.07]	0.51
rs9592482	-0.04	[-0.16, 0.07]	0.48
rs1248860	-0.03	[-0.13, 0.07]	0.57
**All**	**-0.06**	**[-0.17, 0.05]**	**0.31**

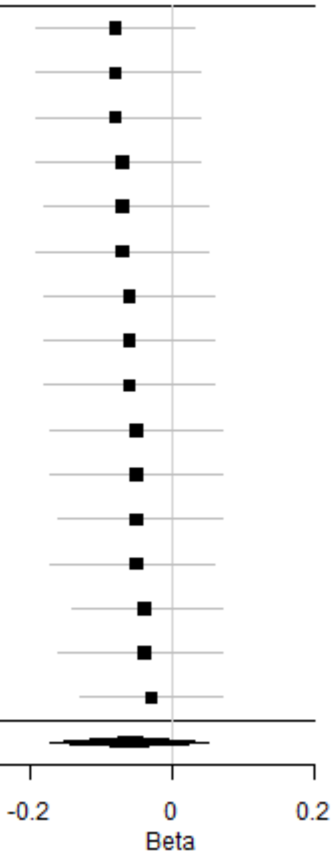

Supplementary table 2: Proportion (%) of individuals with a disorder associated with fatigue across categories of tiredness. Individuals with disorders were extracted from the ICD-10 or the self report codes.

	Not at all	Several days	More than half the days	Nearly every day
Multiple Sclerosis	22.3	43.4	10.6	23.6
Rheumatoid Arthritis	30.3	46.1	9.4	14.2
Inflammatory bowel disease	31.6	43.6	9.8	15.0
Cancer	43.4	42.2	6.5	7.9
Long standing illness	35.0	44.4	8.6	12.0
Depression	20.9	46.8	11.8	20.5
Chronic fatigue Syndrome	11.3	36.7	11.6	40.5
